# Coarse-to-Fine Classification of Road Infrastructure Elements from Mobile Point Clouds Using Symmetric Ensemble Point Network and Euclidean Cluster Extraction

**DOI:** 10.3390/s20010225

**Published:** 2019-12-31

**Authors:** Duo Wang, Jin Wang, Marco Scaioni, Qi Si

**Affiliations:** 1 Department of Information, Beijing University of Technology, Beijing 100124, China; duo.wang@emails.bjut.edu.cn; 2 Beijing Key Laboratory of Traffic Engineering, Beijing University of Technology, Beijing 100124, China; SiQ@emails.bjut.edu.cn; 3 Chinese Academy of Surveying & Mapping, Beijing 100830, China; 4 Department of Architecture, Built Environment and Construction Engineering, Politecnico di Milano, 20133 Milano, Italy; marco.scaioni@polimi.it

**Keywords:** deep learning, Euclidean cluster extraction, point cloud, mobile laser scanning, road infrastructure, symmetric ensemble point network

## Abstract

Classifying point clouds obtained from mobile laser scanning of road environments is a fundamental yet challenging problem for road asset management and unmanned vehicle navigation. Deep learning networks need no prior knowledge to classify multiple objects, but often generate a certain amount of false predictions. However, traditional clustering methods often involve leveraging a priori knowledge, but may lack generalisability compared to deep learning networks. This paper presents a classification method that coarsely classifies multiple objects of road infrastructure with a symmetric ensemble point (SEP) network and then refines the results with a Euclidean cluster extraction (ECE) algorithm. The SEP network applies a symmetric function to capture relevant structural features at different scales and select optimal sub-samples using an ensemble method. The ECE subsequently adjusts points that have been predicted incorrectly by the first step. The experimental results indicate that this method effectively extracts six types of road infrastructure elements: road surfaces, buildings, walls, traffic signs, trees and streetlights. The overall accuracy of the SEP-ECE method improves by 3.97% with respect to PointNet. The achieved average classification accuracy is approximately 99.74%, which is suitable for practical use in transportation network management.

## 1. Introduction

Three-dimensional point clouds obtained from mobile laser scanning (MLS) in road environments have received considerable attention due to the increasing demand for their accurate understanding [1]. Point clouds can provide completeness and a rich level of detail for the objects located on roads. On the other hand, the massive volume of points collected by an MLS system may contain local redundant data that may increase the data volume. This type of data sets may also feature a variable point density and a high number of incomplete structures due to the presence of occlusions [2]. These problems, for example, prevent the directly exploitation of the three-dimensional high-precision map and autonomous vehicle navigation, as described in [3]. Consequently, classification of road infrastructures from such dense point clouds needs to be investigated both theoretically and practically.

The following subsections review relevant works in the literatures that include detection and classification of objects from geospatial data, including either images and point clouds as data sources. Briefly speaking, the works are grouped into rule-feature-based and deep-learning-based methods.

### 1.1. Rule-Feature-Based Classification Methods

In early research works, a set of predefined discriminant rules were used to extract a single object (e.g., [4,5]). These rules are effective but show limitations when adopted in complex environments which may often contain considerable uncertainty and outliers [6,7]. To classify multi-objects, machine learning techniques with leveraging priori knowledge have been proposed [8], such as random forest (RF) [9], support vector machine (SVM) [10], decision tree [11] and Euclidean cluster extraction (ECE) [12].

Interesting applications of RF are briefly introduced here. Becker et al. used RF and gradient boosted trees to train the classifier by considering a multi-scale pyramid with decreasing point densities, combined with HSV colour values of aerial photogrammetry data [13]. Road curbs and markings in MLS data are detected by a binary kernel descriptor and RF classifiers [2]. Niemeyer et al. integrated an RF classifier with the conditional random fields method, and demonstrated a 2% increase in the overall classification accuracy with contextual features considered [14]. The limitation of this classification method is the over-smoothing problem wherein both small and large objects can be easily wrongly classified [15]. Other applications also demonstrated the active effects of RF in various scenarios (e.g., [16,17]).

An SVM approach with geometrical and contextual features was proposed to extract 3D objects in urban scenes [18]. In order to classify images, an SVM-based edge-preservation multi-classifier relearning framework was developed to classify the high-resolution images and achieve highly accurate interpretation [19]. Xiang et al. segmented the initial point clouds, and then extracted features with three popular classifiers—SVM, RF and extreme learning machine (ELM) [20]. On an average, both SVM and RF classifiers reached similar precisions and recall rates in classifying grounds, trees and buildings. Other similar applications also reported desirable performances of SVM [21,22]. However, these classifiers often label each point independently from their local features and do not consider the semantic labels of the neighbouring points, which often leads to noisy results, especially in complex scenes [23].

Given a set of points, the points within each cluster are similar to each other and the points from different cluster are dissimilar. On the basis of this concept, the Euclidean cluster extraction (ECE) adopts a 3D grid subdivision of the space that is fast to build and useful for situations where either a volumetric representation of the occupied space is needed, or the data in each resultant 3D grid can be approximated with a different structure [12]. This strategy could cope effectively in the case of road infrastructure, which may be segmented into clusters based on the Euclidean distance.

### 1.2. Deep-Learning-Based Classification

Recently, deep learning network techniques have been successfully applied to data segmentation and classification. Basically, the deep learning networks are composed of multiple processing layers, with the aim of learning the representations of data with multiple levels of abstraction. Convolutional neural networks (CNNs) [24] are the primary architecture that has been used in deep learning methods for segmenting and classifying objects [25,26]. In applications such as the classification of individual tree species, depth images are learned by a CNN to describe the characteristics of each species [27]. For detecting multi-class geospatial objects, a weakly supervised deep learning method was proposed by leveraging pair-wise scene-level similarity to learn discriminative convolutional weights, and by using pairwise scene-level tags to learn class-specific activation weights [28]. In [29], an automated framework combining CNN and three-dimensional point-cloud features is applied to aerial imagery for the detection of severe building damages caused by disasters. These methods focused on the classification/extraction of objects in 2D aerial or satellite images.

Based on CNN, a fully convolutional network (FCN) takes inputs of arbitrary size and produces outputs of the corresponding size. It introduces skip connections as a way of fusing information from different depths, which correspond to different image scales [30]. The U-net [31] concatenates feature maps from the contracting path. It combines low-level details and high-level semantic information, and achieved good performance on biomedical image segmentation. The SegNet [32] consists of an encoder network and a corresponding decoder network, which maps the low-resolution encoder features to all input-resolution features for a better segmentation accuracy. The DeconvNet [33] fused detail and semantic features for segmentation purpose. The up-sampling of DeconvNet is similar to the SegNet.

In two-dimensional images, the elementary radiometric information is organised in regular grid of pixels where spatial relationships among them can be caught by using moving filtering windows. However, three-dimensional point clouds are unorganised point structures in which the density maybe uneven [34]. To overcome this drawback, the point clouds are transformed into regular three-dimensional voxels or two-dimensional raster structures before feeding them to a deep learning network. Voxel-based (e.g., ShapeNet [35]), multi-view-based (e.g., Multi-view CNN [36]) and point-based CNN (e.g., PointNet [37]) techniques are popular networks to process 3D data and to extract the features/characteristics of objects based on the CNN techniques.

Some interesting investigations on 3D data segmentation and classification are briefly introduced here. By projecting point clouds into raster data sets, road markings are extracted, classified and completed based on the popular U-net, CNN and generative adversarial network (GAN) networks, respectively [38]. By generating a CNN to leverage a spatially local correlation, PointCNN [39] is proposed to classify multiple benchmark data sets using an χ-Conv operator, which weighs and permutes point clouds. Instead of sigmoid as the activation function, Zhang et al. [40] used a rectified linear unit neural network (ReLu-NN) to speed up the convergence and reduce the number of neurons to avoid over-fitting airborne laser scanning data. KD-networks are designed for three-dimensional data recognition with open indoor data [41]. In the case of high-resolution three-dimensional data, OctNet is presented by hierarchically partitioning the space with unbalanced octrees [42].

A multi-layer perception (MLP) can be viewed as a logistic regression wherein the input is first transformed using a non-linear learnt transformation, which then projects the input onto a space where it becomes linearly separable. This intermediate layer is referred to as a “hidden” layer. A single hidden layer is sufficient to make MLPs a universal approximator. In the case of very deep network with hundreds of layers, ResNet [43] is proposed for solving the gradient vanishing problems by using residual blocks. Although slightly better than the approach of directly processing unsorted points, the direct application of MLP on unsorted point clouds does not perform well [37]. 

Instead of transforming irregular point clouds to voxel grids, Qi et al. [37] directly exploited the point clouds for segmentation and classification by designing a PointNet, which is permutation and transformation invariant. Evaluated on modelNet40 [36], the PointNet is robust and performs at the same level as, or, in some cases, even better than, other state-of-the-art solutions. Interesting applications are demonstrated, for example, in learning local normal and curvatures [44] and in segmentation based on sections along the road [45]. Later, Qi et al. [46] introduced a PointNet++ network to cope with the uneven point cloud density. This network has been applied to the classification of coniferous and deciduous trees [47]. VoteNet demonstrates significant improvements in object detections and the authors suggest to apply in downstream point cloud segmentation [48].

The PointNet and its variants were tested in indoor environments and provided reliable results, offering a new option of being transferred to other domains [37]. However, the PointNet and PointNet++ process each point in the local point set individually and does not extract the relationships, such as distance and edge, between the point and its neighbours [49]. This may result in problems when classifying small objects and neighbouring objects that lie within a short distance from one another. 

### 1.3. Motivations and Main Contributions

Deep learning networks need no prior knowledge to classify multiple objects, but often may generate a certain amount of false predictions. Classic segmentation/clustering methods, however, often involve leveraging a priori knowledge and require less data, but may lack generalisability in comparison to deep learning networks. This study proposes a symmetric ensemble point (SEP) network based on PointNet [37] for coarse classification of infrastructure elements along roads (including road surfaces, buildings, trees, walls, streetlights and traffic signs) from point clouds obtained with MLS. In addition, we use a Euclidean cluster extraction (ECE) method to refine the prediction of points that previously have been incorrectly classified. The proposed approach takes advantage of PointNet’s ability to directly process raw point clouds, the ensemble method’s ability to enhance model robustness and the Euclidean distance clustering’s ability to classify neighbouring points at a fine scale. The main contributions of this paper are as follows:

- A novel road infrastructure classification method is developed by combining an SEP network that directly classifies massive point clouds and an ECE method which has potential to adjust falsely predicted points;
- To enhance the robustness of the network and to avoid over-fitting by introducing an ensemble method that trains sub-models and casts them into four bootstrap sample sets; and
- To validate the proposed method with public and an experimental data set.

Section 2 introduces the proposed model for a coarse-to-fine classification of infrastructure elements. Section 3 provides details of the public and experimental data. Section 4 shows the implementation details and discusses the classification results. Eventually, a summary of the proposed method for infrastructure classification from point clouds is presented in Section 5.

## 2. Materials and Methods

**Figure 1:**
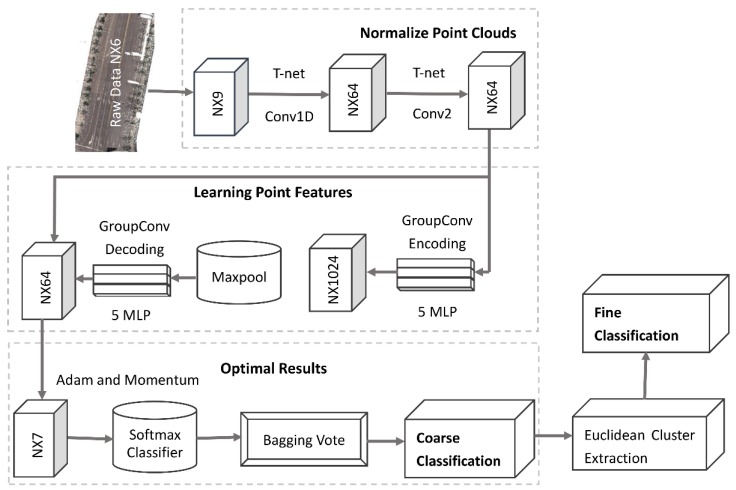
Workflow of the symmetric ensemble point (SEP)-Euclidean cluster extraction (ECE) method, where the left part shows the coarse classification network and the right part shows the refining method to adjust falsely predicted points.

The proposed SEP-ECE framework for a coarse-to-fine classification of multiple road infrastructure elements from unstructured and unordered point clouds includes the following components (Figure 1):

- Coarse classification with an SEP network by normalisation of raw point clouds and extraction of object features based on an encoding and decoding network (Section 2.1);
- Application of an ensemble method for optimising the classification results (Section 2.2).
- Fine classification with the ECE method (Section 2.3) for the adjustment of false predictions that often occur when classifying objects with similar local features, such as traffic signs, streetlights, trees, buildings and walls.

### 2.1. Encoder-Decoder with Normalised Point Clouds

Regarding the point-level labelled data, multi-object classification from point clouds can be regarded as a semantic segmentation problem. MLS data usually contains three-dimensional coordinates (x,y,z) and colour (r,g,b)/intensity (i) information [50]. In order to extract point features in more details, six information channels (x,y,z,r,g,b) from the point cloud are introduced as the input data, although the model may also support other channel combinations such as (x,y,z) or (x,y,z,i). To achieve rotation invariance of the unstructured point cloud, it is necessary to normalise the input data and their colours using a **P** matrix of size N×9, where N is the number of points and the nine columns include the three-dimensional point coordinates, their RGB values, and corresponding 3D coordinates in local coordinate systems.

A sub-net called T-net [37] trains a 9×9 coordinate transformation matrix **A** using **P** matrix as the input. By multiplying **P** and **A**, a 64×64 feature rotation matrix is also obtained through training. This operation results in the normalisation of point coordinates (x,y,z) and colour information. For successful optimisation, L2 regularisation is performed to avoid over-fitting the network, and matrix A is restricted to closely represent an orthogonal matrix. The minimisation is performed on the following Function [37]:

(1) $$Loss_{total}=Loss_{softmax}+{\left|\left|I-AA^{T}\right|\right|}^{2}$$

where I is the unit matrix; $Loss_{total}$ is the total loss function for the optimisation; ${\left|\left|I-AA^{T}\right|\right|}^{2}$ is the regular penalty function, to restrict the T-net transformation; $Loss_{softmax}$ is the loss function of the Softmax layer [51], and the cross-entropy Loss is used to measure the difference between the predicted result and the label.

Based on the normalised point clouds, learning object features are related using feature encoding and decoding method. A series of MLP can be seen as dimensional maps, from low to high level [31]. With a group of MLPs, the features of point clouds are mapped onto a higher dimensional space to be classified, which is sparser and independent. Normally, more high-level layers may extract more features from the point cloud, but the computing time would quickly increase. Thus, five MLP layers (64, 128, 256, 512, 1024) are tested and selected to increase the feature dimensions of the point cloud. In lower dimensions, the network attempts to learn some local features, while in the higher dimensions, the network assembles those local features into global features. Then, a symmetric function (max pooling) is used to find the edge information of those features. At this stage, sub-sampled features are obtained in the higher dimensional space (1024).

To increase local features and avoid the gradient vanishing problem, we concatenate the original normalised features with global feature maps. As with the feature encoding part, the decoding part also includes five MLP layers (512, 256, 128, 128, 64). This architecture reorganises smoother feature maps in low dimensions, performing better during the classification.

In order to make the network converge faster, the Adam algorithm is used to optimise this task [52]. This algorithm adapts a learning rate and quickly brings the parameters closer to an optimal solution. However, in some cases, it may cause the parameters to converge into a very sharp local minimum. Hence, in order to make the model flatter and robust, a momentum method [53] is applied to finely tune the parameters. The details of the designed point cloud normalisation and MLP are given in Table 1.

### 2.2. Optimal Ensemble Method

This sub-section is related to a Softmax classifier and an ensemble method. In order to obtain the class probability of each candidate, a cross-entropy is set as the loss function. This in turn helps to measure the difference between the predicted results and the labels.

(2) $$Y_{i}=softmax\left(X_{i}\right)=\frac{\exp\left(x_{i}\right)}{\sum_{j}\exp\left(x_{j}\right)}$$

(3) $${\mathrm{Loss}}_{softmax}=H_{Y_{gt}}\left(Y\right)=-\underset{i}{\sum }Y_{gt_{i}}\log\left(Y_{i}\right)$$

where $X_{i}$ is the output of the last perception of the i-th class (i is ignored in the following), Y is the output of the Softmax layer, and $Y_{gt}$ is the probability distribution of labels (i.e., the ground truth). The Softmax layer converts the value from the last perceptron into a probability distribution. An end-to-end classifier f(Q) is used to output the label $L_{i}=f\left(p_{i}\right)$ of the point $q_{i}$ in the point cloud Q. The model outputs a series of scores that indicate the probability for each candidate class. The label of this point is the maximum score of the series.

**Figure 2:**
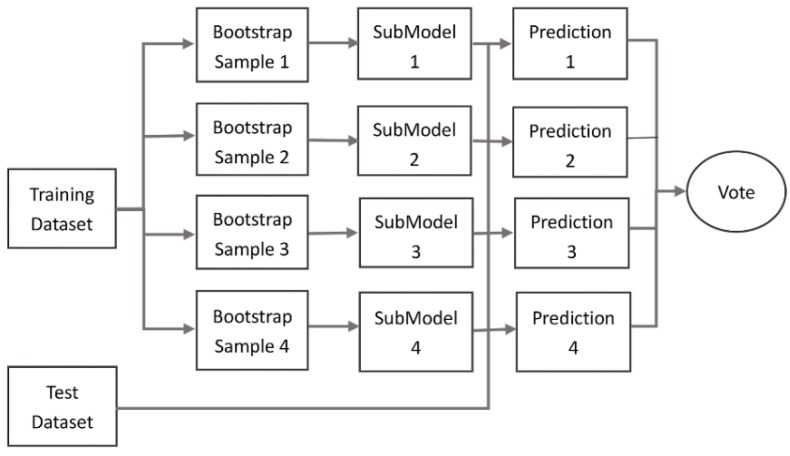
The illustration of an ensemble method.

An ensemble method (Figure 2) is used to obtain a more robust model and to avoid over-fitting of the network. Based on a bagging strategy, the training samples are sub-sampled and constructed into four bootstrap sample sets for training four sub-models. Each sub-model outputs the classification result of the test sample in the form of a vote. The class with the largest number of votes is chosen as the prediction result of the point $q_{i}$ [54,55].

During the bagging vote, the weight of each sub-model is set as $\frac{1}{m}$, where m is the number of sub-models (f). All expectations in $E\left(f_{k}\right)$, where k denotes the k-th sub-model, are approximately equal, so that the entirety model expectation E(F) can be simplified as:

(4) $$E\left(F\right)=E\left(\overset{m}{\underset{k}{\sum }}\gamma_{k}*f_{k}\right)=\overset{m}{\underset{k}{\sum }}\gamma_{k}*E\left(f_{k}\right)=\gamma *\overset{m}{\underset{k}{\sum }}E\left(f_{k}\right)=\frac{1}{m}*m*\mu =\mu$$

(5) $$\begin{array}{l} Var\left(F\right)=Var\left(\sum_{k}^{m}\gamma_{k}*f_{k}\right)\\ =Cov\left(\sum_{k}^{m}\gamma_{k}*f_{k},\sum_{k}^{m}\gamma_{k}*f_{k}\right)\\ =\sum_{k}^{m}\gamma_{k}^{2}*Var\left(f_{k}\right)+\sum_{k}^{m}\sum_{p\neq k}^{m}2*\rho *\gamma_{k}*\gamma_{p}*\sqrt{Var\left(f_{k}\right)}*\sqrt{Var\left(f_{p}\right)}\\ =m^{2}*\gamma^{2}*\sigma^{2}*\rho +m*\gamma^{2}*\sigma^{2}*\left(1-\rho \right)\\ =m^{2}*\frac{1}{m^{2}}*\sigma^{2}*\rho +m*\frac{1}{m^{2}}*\sigma^{2}*\left(1-\rho \right)\\ =\sigma^{2}*\rho +\sigma^{2}*\frac{\left(1-\rho \right)}{m} \end{array}$$

where $\gamma_{k}$ is the weight of each sub-model; *F* is the overall model; $\sigma^{2}$ is the variance of each sub-model due to the introduction of bootstrap sample strategy and identically distributed samples; and ρ is the correlation factor of the sub-models. The variance of the whole model is computed from each sub-model and its weight $\gamma_{k}$ in the bagging vote. If each sub-model has an equal weight (γ), the variance of the whole model is computed from the average results of each sub-model. Equation (4) shows that the expectation of the whole model is approximately equal to the sub-model expectation. Equation (5) certifies that the variance of the whole model is equal to or less than the variance of each sub-model, because the variance of the mean model decreases when the number of sub-models increases. In a special case of Equation (5), when ρ=1, the variance of the sub-model is equal to the one of the whole model. In other words, the performance of the whole model relies on the performance of each sub-model.

Hence, to ensure the effectiveness of the whole model, the sub-model needs to have enough capability for classification. In addition, the introduction of bagging vote reduces the random errors in the stage of data training. It enhances the generalisation ability and improves the accuracy of the network.

### 2.3. Refining Classification with ECE Method

The SEP network may correctly classify most objects, such as road surfaces and buildings. However, a small number of points are falsely predicted when they have similar features, for example, in the case of pole-like structures or planar surfaces. To correct these points, an ECE method is implemented [12] under the assumption that the point clouds of neighbouring objects have a distance between each other.

A simple Euclidean data clustering approach is applied by creating a 3D grid subdivision of the space using a k-d tree data structure [56]. We define a cluster of points $S_{u}=\left\{q_{u}\in Q\right\}$ to be a distinct from cluster $S_{v}=\left\{q_{v}\in Q\right\}$ if:

(6) $$min{\left\|q_{u}-q_{v}\right\|}_{2}\geq d_{th},$$

where $d_{th}$ is an imposed maximum distance threshold and Q is the input dataset from the results of SEP network. The above equation states that if the minimum distance between two sets of points $q_{u}\in Q$ and $q_{v}\in Q$ is larger than a given distance value, then the points in $q_{u}$ are set to belong to an object cluster $S_{u}$ and the ones in $q_{v}$ to another distinct object cluster $S_{v}$. In the following, the algorithm to cluster points is described by using approximate nearest neighbour queries:

(1) a k-d tree structure for the input point cloud dataset Q is created;
(2) an empty list of clusters C and a queue of the points that need to be analysed S is set up;
(3) the following steps are run per every point $q_{u}\in Q$:
  - $q_{u}$ is added to the current queue S;
  - The following operations are executed per every point $q_{u}\in Q$:
    ■ search for the set $Q_{w}^{u}$ of point neighbours of $q_{u}$ in a sphere with radius $r_{th}<d_{th}$;
    ■ for every neighbour $q_{w}^{u}\in Q_{w}^{u}$*,* if the point has not been processed yet, it is added to S;
  - when all points in S have been processed, add S to the list of clusters C and reset S to an empty list.
(4) the algorithm terminates when all points $q_{u}\in Q$ have been processed and are assigned to a cluster;
(5) for a cluster $S_{C}$ in ***C*** do:
  - all the classes in this cluster are counted and the main class is selected as the representative class of this cluster;
  - the properties of the cluster are checked with prior knowledge, such as bounding boxes, density, gravity centres, heights.

The proposed SEP network coarsely classifies points into groups; while the application of the ECE method refines the classification results, especially to detect and revise the falsely predicted points that may be in small and neighbouring objects.

## 3. Experimental Data

In this experiment, we have used two datasets: (1) the publicly available Stanford 3D semantic parsing data set for comparing the networks’ performances with respect to state-of-the-art solutions; and (2) the experimental data set collected by Leica Pegasus 2 MLS in a road environment. These data sets are addressed in the following as Data Set 1 and 2, respectively.

### 3.1. Stanford 3D Semantic Parsing Data Set 1

The publicly available Stanford 3D semantic parsing data set [57] concerns building indoor data collected by Matterport scanners in six areas including 271 rooms. Each point is annotated with one of the semantic labels from 13 categories (ceiling, floor, wall, column, beam, window, door, table, chair, bookcase, sofa, board and clutter). Although the target of our investigations is the classification of road infrastructures, the use of this indoor data set gives a chance to compare the performances of the symmetric point (SP) network against the results obtained by the PointNet approach in [37], which may be considered as a state-of-the-art solution.

### 3.2. Experimental Road Data Set 2

**Figure 3:**
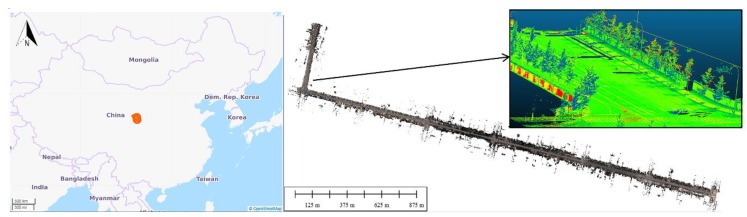
From left to right: Geographical location of Data Set 2 (from OpenStreetMap); and planimetric view of the road point cloud collected by the mobile laser scanning (MLS), with a zoom-in of the point cloud coloured with laser intensity values.

**Figure 4:**
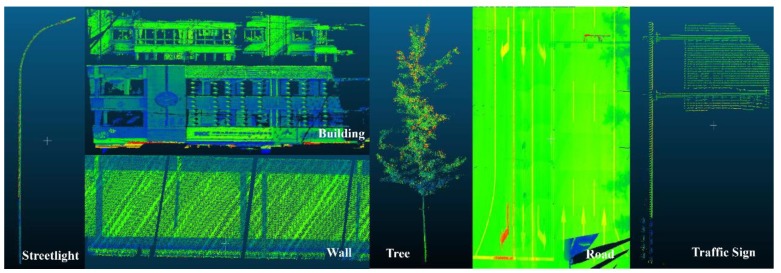
Example of six classes (from left to right: streetlight, building, wall, tree, road, traffic sign) identified in the classification experiment based on Data Set 2. Data are coloured with laser intensity.

A data set collected by Leica Pegasus 2 MLS in Jianning East Road (LanZhou, China) has been adopted to validate the proposed coarse-to-fine classification method (see Figure 3). The Leica Pegasus 2 mainly includes a laser scanner, eight cameras and a triple-band GNSS. The test road is 500 m apart from the Yellow River. The length of the test road is approximately 3.5 km, with 273.52 million points having associated 3D spatial coordinates and RGB information. Regarding the point density on the ground, the average point spacing was approximately 20 cm and 6 cm in the driving and perpendicular directions, respectively. Point clouds have been manually annotated into six classes (buildings, road surface, trees, walls, traffic signs, streetlights) and clutters (Examples of each class is shown in Figure 4).

## 4. Implementation Details, Results and Discussion

The SEP network has been coded by in Python 3.5 and Tensorflow 1.0. A computer powered by one GPU (Intel Xeon E5-2620, 32 GB RAM, Nvidia GeForce GTX 1080Ti from Gigabyte Technology Co. Ltd, New Taipei City, Taiwan) has been used to run the code. The ECE model has been performed with C++ language with point cloud library (PCL).

Precision, accuracy and recall are used to evaluate the quality of the obtained classification outputs:

(7) $$\begin{array}{l} Precision=TP/\left(TP+FP\right)\\ Accuracy=\frac{TP\;+\;TN}{TP\;+\;TN\;+\;FP\;+\;FN}\\ Recall=\frac{TP}{TP\;+\;FN} \end{array}$$

where TP, FP, TN and FN denote the numbers of true positives, false positives, true negatives and false negatives, respectively. The overall accuracy has been computed from the confusion matrix for quantitative assessment.

### 4.1. Comparative Analysis of Data Set 1

Five areas from the Stanford 3D semantic parsing data set have been selected to train the deep learning network and another independent area has been adopted for testing the model performance. The overall accuracy of PointNet and the symmetric point (SP, without using the ensemble method) network have resulted in 77.24% and 79.81%, respectively (see Table 2). The overall accuracy has improved by 2.57% with the help of symmetric MLP with respect to PointNet. This improvement illustrates the validation of the SP network. Based on this SP network and the ensemble method, the coarse classification is proposed and tested on Data Set 2. The difference in the selection of the training and testing data sets caused the accuracy of the PointNet computed here to be different from the results published in [37].

### 4.2. Implementation Details and Classification Results of Data Set 2

#### 4.2.1. Implementation Details

The experimental Data Set 2 is split into 12 areas. The training and testing samples have been selected in a 5:1 proportion. Ten areas (1–9, 11) have been used to train the model. This task has required approximately one processing day. Two remaining areas (10 and 12) have been used to check the performance of the model. Processing has taken approximately one hour.

A batch has been randomly selected from the whole data set. Each batch consisted of 24 blocks. In each block, 4096 points have been used for training and testing the network. Each point is represented by a 9 dimensional vector composed of x, y, z, R, G, B and 3D normalised coordinates in the block. The default parameters for training the ten areas (1–9, 11) have been defined with 50 maximum epochs to run, 0.001 learning rate, 0.9 momentum, 300,000 decay step, and 0.5 decay rate. These training parameters were tested to help the network converge rapidly in our device. Meanwhile, the proposed five MLP layers efficiently used the 11 GB graphics memory of the Nvidia GeForce GTX 1080Ti graphic board. 

Based on the results of coarse classification with the SEP network, the ECE model is then applied to revise the false predicted points. The default parameters of cluster tolerance and minimum cluster size are 40 cm and 8000 points, respectively. Furthermore, height differences between streetlights and traffic signs are also used to distinguish among pole-like objects.

#### 4.2.2. Coarse Classification Results with SEP Network

**Figure 5:**
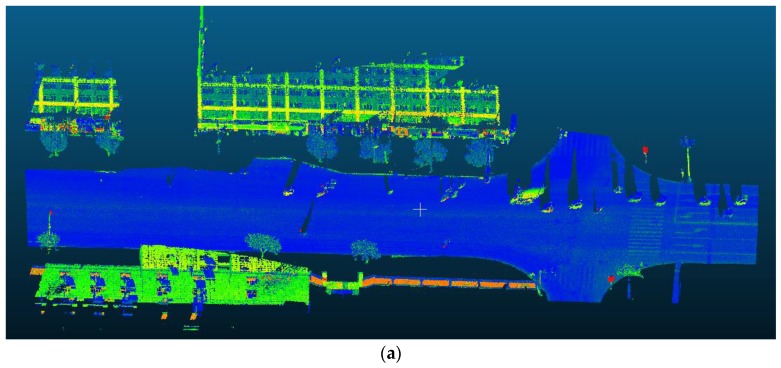

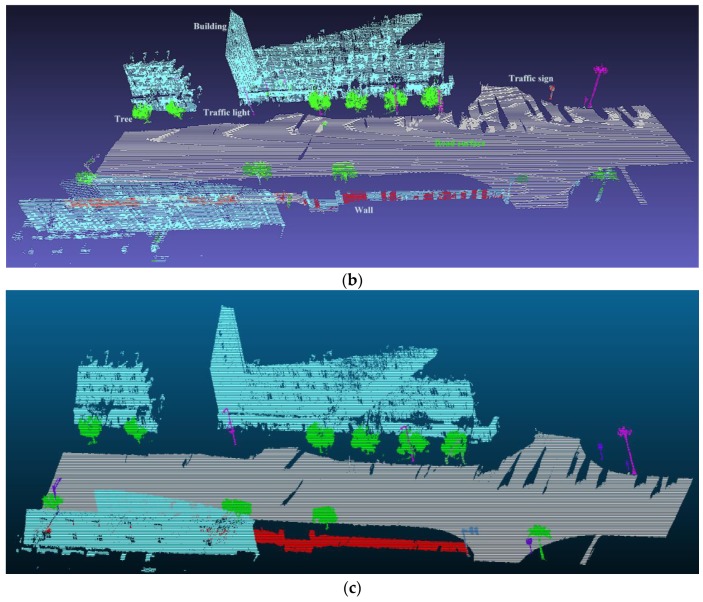
(**a**) view of raw data, (**b**) coarse classification and (**c**) coarse-to-fine classification results of testing area 10. Grey colour denotes the road surface; green colour is the trees; blue colour denotes the building; pink means the streetlight, red colour is the wall and the purple pole-like structure is the traffic sign.

**Figure 6:**
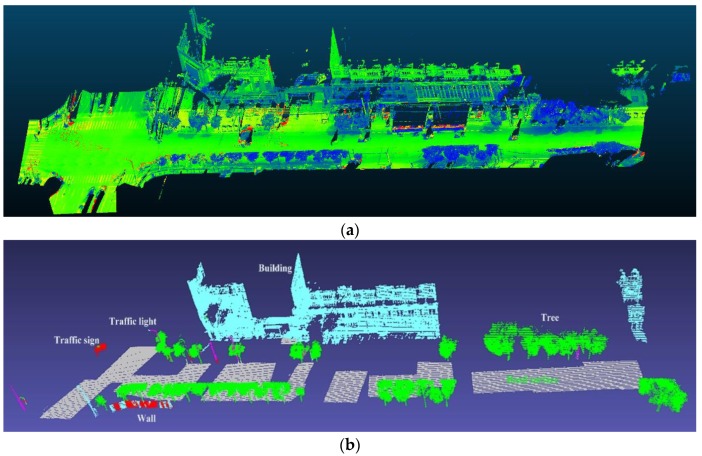

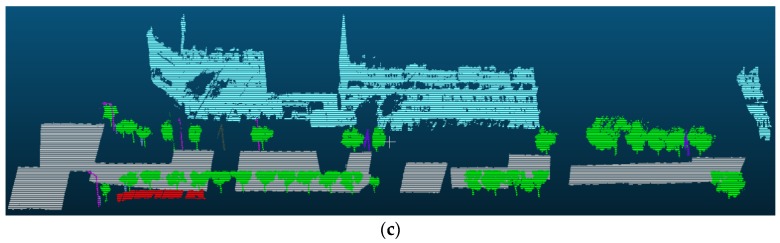
(**a**) view of raw data, (**b**) coarse classification and (**c**) coarse-to-fine classification results of testing area 12, where grey colour denotes the road surface; green colour is the trees; blue colour denotes the building; pink means the streetlight, red colour is the wall and the purple pole-like structure is the traffic sign.

The classification results of the testing data are summarized in Table 3, Figure 5 (testing area 10) and Figure 6 (testing area 12), where the visualisation of raw data is shown on the top of the figure, while the classification results are displayed at the bottom. Blank areas at the bottom of Figure 5 and Figure 6 are due to clutters, and clipped incomplete structures which have been filtered out from the data set to clearly highlight the classification results. Indeed, the clipping of cars and outliers on the road surface has resulted in the discontinuity of the road surface.

As shown in Table 3 and Figure 5 and Figure 6, the SEP network correctly classifies most road infrastructure elements and extracts common objects, such as the road surfaces, buildings and trees. Traffic signs and walls are sometimes conflated, as shown at the bottom of Figure 5, because the local characteristics of both types of items are similar to a plane. Tree trunks and traffic signs sometimes are also mixed due to similar geometric shapes (both have a cylindrical surface). The same confusion also appears in small areas between walls and buildings. Different scales of point features may cause object misclassification. On the other hand, larger variations of object numbers also influence the classification results. The next fine-scale classification step processes these falsely predicted points which commonly exist in deep learning classification.

#### 4.2.3. Refining Classification Results with SEP-ECE Method

The fine-scale classification is based on the results from the SEP network. As demonstrated in Figure 5 and Figure 6, a small number of points are falsely predicted. For example, a part of a wall is wrongly classified as a road surface and a part of a traffic sign as tree. The classified points are then fed into the ECE model. The coarse-to-fine classification results with the SEP-ECE method are shown in Figure 5 and Figure 6. The falsely predicted points from the SEP network are revised with the ECE model, especially as far as walls, trees, traffic signs and streetlights are concerned.

**Figure 7:**
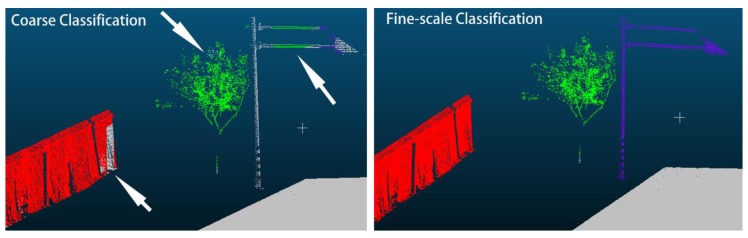
Example on somehow points that were predicted incorrectly with the SEP network are detected and adjusted during the fine-scale classification with the ECE model. The white arrows on the left image point out the misclassified points.

The examples shown in Figure 7 demonstrate that most of the falsely predicted points are adjusted to their true classes (i.e., trees, walls and traffic signs). This refining processing solves problems with points that were misclassified due to the similarity between respective features.

#### 4.2.4. Accuracy, Precision and Recall of the SEP-ECE Method

Regarding accuracy, precision, and recall, we compared the results among the SEP-ECE method, SEP network, SP network, and the PointNet with independent validation data from Data Set 2 (see Table 4, Table 5 and Table 6). These results show that the proposed SEP-ECE method preformed best among the comparisons with SEP network, SP network and PointNet. The mean accuracy of our model is 3.97% higher with respect to PointNet. Both of the precision and recall were also improved with the proposed coarse-to-fine classification method.

When comparing the proposed SEP network and PointNet, the local characteristics of roads and walls are similar in geometric shape, so the deeper layers of perceptron may cause the precision of the road surfaces and recall of the walls to be lower than the ones obtained with PointNet. There are three reasons for the imbalance in the precision and recall of traffic signs and streetlights between the two methods: (1) asymmetric samples (e.g., between roads and traffic signs); (2) little numbers of points in the case of small objects (e.g., traffic signs and streetlights); and, (3) the relatively complex decoder structure raising the criteria of specific classes.

Fuzzy boundary problems that commonly existed in classification with deep learning networks are processed by adding a priori knowledge to the fine-scale classification. The performance of the ECE model efficiently solved the fuzzy boundary points and revised the falsely predicted points into correct groups, especially the objects with small sizes.

**Figure 8:**
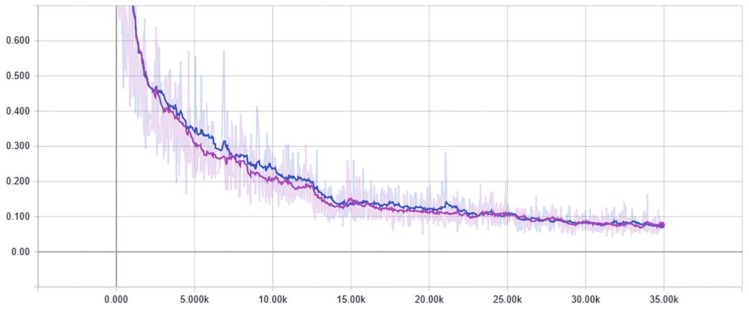
Results of the optimisation related with iterative times and loss, where purple line indicates the results of PointNet and the blue line indicates the results from the proposed SEP network.

Although the losses of the SEP network at the beginning are slightly higher than PointNet, both of the methods almost converge at the same time as shown in Figure 8. After 15,000 iterations, the model tends to stabilise. In other words, the SEP network was slightly more accurate than the PointNet, but with a time cost that is nearly the same.

### 4.3. Discussion

The deep learning network (SEP network) developed with the purpose of coarsely classifying MLS data in the road environment has two key modules: (1) a symmetric encoding–decoding network combined with the max pooling layer as a symmetric function, a local and global information structure, and two joint alignment networks, and (2) an ensemble method to optimise the results from a sub-sample and avoid over-fitting of the network. 

Five MLPs in the proposed SEP network are deeper than the one of the classic PointNet with two MLP layers [37]. The usage of five MLPs obtained both global and detailed/local features in higher dimension. To guarantee the quality of classification, the contact layer is proposed to combine both global and local features. At the same time, the contact layer guarantees the correct transfer of the object characteristics. The symmetric structure of the encoding-decoding network refines the 9-dimension features with higher dimension features. This helps the learned features to merge more local point features, and it improves the classifying ability to distinguish multiple objects.

In the ensemble method, each sub-model outputs the classification result of the test sample in the form of a vote, and the highest vote is selected as the prediction result. The usage of the bagging vote method improves the robustness and avoids over-fitting of the network. The performed ensemble method reduced the random errors of objects in the stage of data training, and increases the generalisation of the network. The average performance of the Bootstrap strategy is equal or improves the performance of the model without using bootstrap. 

Most of the points are correctly classified on the basis of SEP network. However, less common objects, or elements that share similar features, are sometimes misclassified and object boundaries may be fuzzy. This phenomenon is quite common in classification methods based on deep learning networks, because it is difficult to evaluate if the deep neural network has sufficiently accounted for the geometric relationships between neighbouring points. The ECE model, however, focuses on the distance relationships between points. As most of the road infrastructure elements are separated in space, the ECE model adjusts the falsely predicted points and improves the accuracy, precision and recall of road infrastructure classification. This is demonstrated in our experiment.

From the results of publicity Data set 1, the overall accuracy was 79.81% from independent validation data, and has improved by 2.57% with the help of symmetric MLP with respect to the method given by PointNet [37]. Furthermore, compared with Data Set 2, the overall accuracy of the SEP-ECE method was 99.74% from independent validation data, and has improved by 3.97% with respect to PointNet [37]. Both precision and recall were also improved with the proposed coarse-to-fine classification method. The proposed network can be applied to other scenarios, because the basic network comes from the classic deep learning network that does not need to manually design features. This means that if the data set is changed, the network could also automatically learn new features. 

The reasons for the lower accuracy with Data Set 1 compared to Data Set 2 are: (1) the data volume of Data Set 2 is larger than the Data Set 1. More data trained in supervised deep learning networks often means better accuracy in the classification results; (2) main objects (e.g., road surface vs. trees) in Data Set 2 are easier to be classified in space than indoor objects in Data Set 1; and (3) the introduction of the ensemble method and the ECE model improves the classification accuracy of small objects (e.g., streetlights).

## 5. Conclusions

In this paper, a coarse-to-fine classification method of mobile point clouds is proposed for coarsely classifying road infrastructures with symmetric ensemble point (SEP) network and refining the classification results with Euclidean cluster extraction (ECE) model. The core contributions of our method include coarse classification with the SEP network by using a symmetric function to extract different scales of point features and voting an optimal sub-sample with the ensemble method, and fine-scale classification by using the ECE model to adjust the false predicted points. The SEP network learns more local features and enhances the robustness of the network; the ECE model efficiently solves the fuzzy boundary problems that commonly existed in classification with deep learning networks.

Both publicly available data and experimental data sets were adopted to check the ability of the proposed SEP-ECE method against state-of-the-art techniques. Compared with publicity Data Set 1, the overall accuracy has improved by 2.57% with the help of symmetric MLP with respect to the method given by PointNet [37]. Compared with the experimental Data Set 2, the overall accuracy of the SEP-ECE method improves by 3.97% with respect to PointNet [37]. The overall accuracy from independent validate data was 99.74% in the classification of road infrastructures (including buildings, road surfaces, trees, walls, traffic signs and streetlights). The results show that the proposed method efficiently improves the classification accuracy, and enhances the recall ability of classifying multi-objects with MLS technology.

The limitations and future work are: (1) annotations are time consuming when applied experimental data sets; (2) a fixed block size may misjudge the point category around the block boundaries; (3) a separate ensemble branch is suggested to directly solve the problem of hard samples (e.g., streetlights); (4) some challenging scenes need to tested (e.g., interchanges in metropolitan); (5) the proposed road infrastructure detection method should be expanded to wider applications, e.g., [29], and multiple sources of data should be used, e.g., [29]; and (6) if the neighbouring objects overlapped or are closely near to each other, the fine classification step might be less accurate.

## Figures and Tables

**Table 1:** Details of the designed point cloud normalisation and multi-layer perception (MLP) for classification.

Layer Number	Network Components	Layer Name	Properties
1	Point cloud normalisation	Input layer	Input patch size 24×4096×1×9
2		T-net1	Matrix multiply 24×9×9
3		Convolution (Conv.) 1	Number of filters 64; filter size 9×1; ExpandDims 24×4096×64×1
4		Conv.2	Number of filters 64; filter size 1×1 ExpandDims 24×4096×64×1
5		T-net2	MatMul 24×64×64
6	MLP encoder from Conv.3 to Conv.7; MLP decoder from Conv.8 to Conv.12	Conv.3	Number of filters 64; filter size 1×1 ExpandDims 24×4096×64×1
7		Conv.4	Number of filters 128; filter size 1×1 ExpandDims 24×4096×128×1
8		Conv.5	Number of filters 256; filter size 1×1 ExpandDims 24×4096×256×1
9		Conv.6	Number of filters 512; filter size 1×1 ExpandDims 24×4096×512×1
10		Conv.7	Number of filters 1024; filter size 1×1 ExpandDims 24×4096×1024×1
11		MaxPool	MaxPoolGrad 4096×1 ExpandDims 24×1×1024×1
12		Concatenate	Layer 6 and Layer 11 ExpandDims 24×4096×1088×1
13		Conv.8	Number of filters 512; filter size 1×1 ExpandDims 24×4096×512×1
14		Conv.9	Number of filters 256; filter size 1×1 ExpandDims 24×4096×256×1
15		Conv.10	Number of filters 128; filter size 1×1 ExpandDims 24×4096×128×1
16		Conv.11	Number of filters 128; filter size 1×1 ExpandDims 24×4096×128×1
17		Conv.12	Number of filters 64; filter size 1×1 ExpandDims 24×4096×64×1
18	-	Conv.13	Number of filters 7; filter size 7×1 ExpandDims 24×4096×7×1
19	-	Softmax	-

**Table 2:** Comparison of classification accuracy obtained by using PointNet and SP network.

	PointNet [1]	SP Network
Overall accuracy	77.24%	79.81%

**Table 3:** Coarse classification results of Data Set 2 with the proposed SEP network.

	Buildings	Road Surfaces	Trees	Walls	Traffic Signs	Streetlights
Precision	82.66	98.55	77.23	80.17	100	25.00
Recall	96.16	99.95	97.05	47.96	1.47	5.35
Accuracy	96.46	96.46	96.81	96.08	97.93	97.05

**Table 4:** Comparison of the accuracy among SEP-ECE method, SEP network, symmetric point (SP) network and PointNet applied to Data Set 2. The best performances per each individual types of object classes are shown in bold.

Accuracy	Buildings	Roads	Trees	Walls	Traffic Signs	Streetlights	Mean
SEP-ECE	99.43	99.95	99.59	99.74	99.80	99.91	99.74
SEP	96.51	97.03	96.86	96.15	97.93	97.09	96.93
SP	96.46	96.46	96.81	96.08	97.93	97.05	96.80
PointNet	95.66	96.05	95.60	95.61	96.48	95.22	95.77

**Table 5:** Comparison of the precision among SEP-ECE method, SEP network, SP network and PointNet applied to Data Set 2. The best performances per each individual types of object classes are shown in bold.

Precision	Buildings	Roads	Trees	Walls	Traffic Signs	Streetlights
SEP-ECE	97.64	99.96	97.93	96.02	65.98	100
SEP	82.92	98.54	77.26	80.19	100	28.57
SP	82.66	98.55	77.23	80.17	100	25.00
PointNet	78.25	99.30	70.51	72.61	20.00	16.67

**Table 6:** Comparison of the recall among SEP-ECE method, SEP network, SP network and PointNet applied to Data Set 2. The best performances per each individual types of object classes are shown in bold.

Recall	Buildings	Roads	Trees	Walls	Traffic Signs	Streetlights
SEP-ECE	99.13	99.97	99.13	90.15	77.70	55.77
SEP	96.16	99.95	97.16	50.16	1.50	5.27
SP	96.16	99.95	97.05	47.96	1.47	5.35
PointNet	94.41	98.71	92.11	68.28	23.12	11.06
